# Effects of microwave treatment on the stability and antioxidant capacity of a functional wheat bran

**DOI:** 10.1002/fsn3.2230

**Published:** 2021-03-13

**Authors:** Jing Liu, Jinli Zhang, Wentao Wang, Hanxue Hou

**Affiliations:** ^1^ Engineering and Technology Center for Grain Processing of Shandong Province College of Food Science and Engineering Shandong Agricultural University Tai'an China

**Keywords:** antioxidant capacity, free fatty acid, functional wheat bran, lipase, microwave stabilization

## Abstract

A functional wheat bran (FWB) was obtained from wheat grains that were rich in wheat aleurone. The effects of the microwave (MW) power (2.5, 5.0, 7.5, and 10.0 kW) and treatment time (15, 30, 60, 90, and 120 s) on the moisture and free fatty acid (FFA) content, lipase activity, and antioxidant activity of the FWB were investigated. The purpose of this study is to stabilize the FWB against lipid oxidation and rancidity and as much as possible to retain its antioxidant activities. MW treatment significantly decreased the FFA content, moisture content, and lipase activity of the FWB. Moreover, MW treatment significantly increased the total phenolic content (TPC) and antioxidant activity of the FWB without drastically altering its color. MW treatment at 7.5 kW and 120 s was found to be optimal for stabilizing the FWB and increasing its antioxidant activity. The stabilized FWB was proven to be far more stable than the control FWB during storage. Thus, MW treatment is an effective stabilization method for the storage and utilization of FWB. Additional research is needed for the exact mechanism of the decrease of FFA content and increase of antioxidant activity of FWB induced by MW treatment.

## Graphical abstract

Microwave treatment significantly decreased the FFA content and lipase activity and significantly increased the total phenolic content (TPC) and antioxidant activity of the FWB. The stabilized FWB was far more stable than the control FWB during storage. Microwave treatment is an effective stabilization method for FWB storage and utilization.

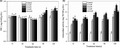

## 1 INTRODUCTION

There is growing evidence that whole grains that are rich in dietary fiber (DF), micronutrients and phytochemicals play an important role in lowering the incidence of cancers, cardiovascular diseases, diabetes, and obesity (Reynolds et al., 2019; Xu et al., 2018). These components are abundant in the germ and bran of wheat kernels (Wang et al., 2016). The replacement of white flour with a percentage of wheat bran is a common way to increase the phytochemical content in wheat products (Blandino et al., 2013). Nevertheless, the incorporation of normal wheat bran into white flour results in deterioration of the processing quality and the final product quality, for example, a lower volume, darker crust and denser crumb texture (Bagdi et al., 2014). Another concern regarding the replacement of white flour with normal wheat bran is the potential risk of contamination with mycotoxins, heavy metals and pesticides (Cheli et al., 2010; Liu et al., 2018).

One of the greatest challenges in the development of whole wheat foods is to minimize these negative effects and as much as possible to improve the sensory acceptability and achieve higher marketability. Preparation of wheat bran fractions that do not contain the outermost layers of wheat kernel was a practical approach since the outermost layers are considered to be responsible for the adverse effects of wheat bran addition. The inner bran fractions that are rich in aleurone are believed to have better functionality than whole wheat bran (Brouns et al., 2012). Thus, bran fractions that are rich in aleurone could be used to develop safe and healthy foods with similar whole grain nutrients and more acceptable color, flavor, and taste than whole grain products (Bagdi et al., 2014, 2016; Blandino et al., 2013; Xu et al., 2020). However, the aleurone‐rich fractions tend to deteriorate due to the hydrolysis and oxidation of the lipids in aleurone and germ (Barnes & Galliard, 1991; Xu et al., 2013). Hence, it is necessary and valuable to stabilize the bran fractions via suitable methods to prolong their shelf life under ambient conditions without significantly damaging the nutritional profile. Although the separation, composition, health aspects, and potential food use of aleurone‐rich bran fractions have been investigated (Brouns et al., 2012), the information of stabilization method and their effects on antioxidant capacity is still scarce.

In microwave (MW) heating, which is a dielectric heating method, food materials absorb microwave energy and convert it into heat, which occurs mainly via dipolar and ionic mechanisms (Chandrasekaran et al., 2013). MW treatment has been widely applied in food processing over the past several decades, for example, in the drying, pasteurization, sterilization, thawing, tempering, and baking of food materials (Zhu et al., 2020). Compared with other heating methods, MW heating is efficient, economical and has a shorter processing time (Chandrasekaran et al., 2013). MW heating has also been widely used to inactivate the lipase of many cereal products, such as germ (Boukid et al., 2018), millet grains (Yadav et al., 2012) and bran (Patil et al., 2016). MW treatment decreased the lipase activity to 14.01% of that of the raw wheat germ, and the acid value of the stabilized wheat germ increased only 6.56% after 60 days of accelerated storage (Xu et al., 2013). In addition, MW treatment has little effect on the nutritional value of rice bran (Patil et al., 2016).

The dielectric property of a food material is the most important factor that determines the effect of MW treatment. The moisture content of a food material plays a key role for its dielectric property since water is a satisfactory absorber of microwaves. In addition to the dielectric property and moisture content, the MW oven design (size and geometry) and MW frequency, along with the density, composition, load, shape and size of the food materials, are also important factors (Chandrasekaran et al., 2013). The most important conditions of MW treatment are MW power and treatment time (Patil et al., 2016).

In this study, the effects of MW treatment conditions (MW power and treatment time) on an aleurone‐rich bran fraction, a functional wheat bran (FWB), were investigated. The FWB was obtained using traditional roller milling after debranning the outermost layers (pericarp layers) of wheat kernels. The storage stability of the FWB, along with the free fatty acid (FFA) content, total phenolic content (TPC), and antioxidant activity after MW treatment, was analyzed.

## 2 MATERIALS AND METHODS

### 2.1 Materials

Wheat grains of a hard white winter cultivar (Jinan 17) were obtained from Jiaoqiao seed station. Soybean oil that had been extracted via a leaching method was purchased from Yihai Grain and Oil Industry Co., Ltd. All other chemicals were of analytical grade and purchased from Chemical Reagent Co., Ltd.

### 2.2 Preparation of the functional wheat bran (FWB)

Wheat grains were cleaned and debranned to remove the outermost layers (approximately 4% of the kernel weight) using a wheat debranner (TPZ‐20). Then, the debranned wheat grains were milled using a laboratory wheat miller (JMFB 70 × 30) to obtain white flour, fine and coarse brans. According to the micronutrient content and antioxidant capacity (unpublished data), the coarse bran was regarded as the FWB. Subsequently, the freshly produced FWB was treated immediately under various MW conditions.

### 2.3 Microwave (MW) treatment of FWB

A microwave dryer (LW‐50HMV‐6X, Shandong Liwei Microwave Equipment Co., Ltd.) with a cooling system was used for the FWB treatment. The belt speed and MW power were controlled by a control panel, and the treatment time was controlled by adjusting the belt speed.

Each FWB sample was placed on the belt, spread evenly to a thickness of 0.3 cm, and treated at a selected MW power (2.5, 5.0, 7.5, or 10.0 kW) for a specified treatment time (15, 30, 60, 90, or 120 s). Immediately after treatment and cooling, the samples were ground and passed through an 80 mesh aperture sieve (180 μm) using a hammer mill (JXFM110, Hangzhou Lvbo Instrument Co., Ltd. China). All samples were stored at a temperature of −18°C in a freezer. Untreated FWB was used as a control.

### 2.4 Moisture content

The moisture contents of the treated FWB and control samples were determined via method 44‐15A (AACC International, 2010).

### 2.5 Color measurement

The color values of the FWB samples were measured using a colorimeter (Model CR‐10 Plus, Keshengxing Instrument Co., Ltd.). The CIE color values were recorded as L* (lightness), a* (redness), and b* (yellowness). The total color differences (*Δ*E) between the control and treated samples were calculated using the equation *Δ*E = (*Δ*L^2^ + *Δ*a^2^ + *Δ*b^2^)^1/2^ (Irakli et al., 2018).

### 2.6 Lipase activity

The lipase activity was determined according to Zhang et al., (2020) with modifications, and the final activity was expressed as mg of KOH consumed by 1.0 g sample on a dry weight basis (d.b.). Briefly, 2.0 g of FWB and 1 ml of soybean oil were placed into a mortar, in which 5 ml of 0.05 mol/L phosphate buffer (pH 7.4) and a small amount of quartz sand had been added in advance. The mixture was ground to form a thin paste and transferred into a conical flask with 5 ml of water. The conical flask was incubated at 30°C for 24 hr. After incubation, 50 ml of an ethanol and ether (4:1) mixed solution was added to the conical flask, and the flask was shaken vigorously. After filtration, the filtrate of 25 ml was titrated with a 0.05 mol/L potassium hydroxide solution, and the volume of the consumed solution was recorded. The same procedure was followed without incubation for the blank control.

### 2.7 Free fatty acid (FFA) content

The FFA content of each of the FWB samples was determined according to a previous report (Wu et al., 2016) with modifications. The value was expressed as mg KOH that was needed to neutralize the free fatty acids in a 100 g FWB sample on a dry weight basis (mg KOH/100 g d.b.).

### 2.8 Total phenolic content (TPC)

To determine the phenolic content, FWB samples were extracted with 60% methanol and centrifuged, the supernatant was filtered through filter paper, and the filtrate was analyzed with Folin Ciocalteu's reagent at 745 nm using gallic as a standard (Irakli et al., 2018). The results were expressed as mg of GA equivalents (GAE) per 100 g of the sample on a dry weight basis (mg GAE/100 g d.b.).

### 2.9 DPPH^+^ radical scavenging capacity assay

Phenolic FWB extracts were mixed with a DPPH^+^ reagent, and the absorbance was measured at 517 nm according to Irakli et al., (2018) with modifications. With 3.8 ml 0.1 mM methanolic solution of DPPH, 0.2 ml extract was reacted. After 1 hr, the absorbance at 517 nm was recorded. The percentage of the scavenging effect was calculated as follows:

$$E_{\text{DPPH}}\left(\%\right)=\left[(1‐\left(A_{S}‐A_{R}\right)/A_{O}\right]$$

where E_DPPH_ is the DPPH radical scavenging capacity, A_S_ is the absorbance of the mixture of 0.2 ml FWB extract and 3.8 ml 0.1 mM DPPH, A_R_ is the absorbance of the mixture of 0.2 ml FWB extract and 3.8 ml methanolic solution, and A_O_ is the absorbance of the mixture of 3.8 ml 0.1 mM DPPH and 0.2 ml 60% methyl alcohol.

The results were expressed as mg Trolox equivalents per 100 g of sample on a dry weight basis (mg TE/100 g d.b.)

### 2.10 Accelerated storage test

An accelerated storage test was conducted in a constant‐temperature oven at 45°C for 4 weeks. The FWB that had been treated under optimal treatment conditions and the control were packed in aluminum foil bags (50 g each). The moisture and FFA contents were assayed at 1‐week intervals.

### 2.11 Statistical analysis

All experiments were conducted in triplicate. The results are reported as the mean value ± the standard deviation and subjected to one‐way analysis of variance, followed by least significant difference calculations, to assess any differences between the group means using the SPSS 20.0 software package. Significance was defined at *p* <.05 by using Duncan's test.

## 3 RESULTS AND DISCUSSION

### 3.1 Effect of MW treatment on the moisture content of the FWB

**FIGURE 1:**
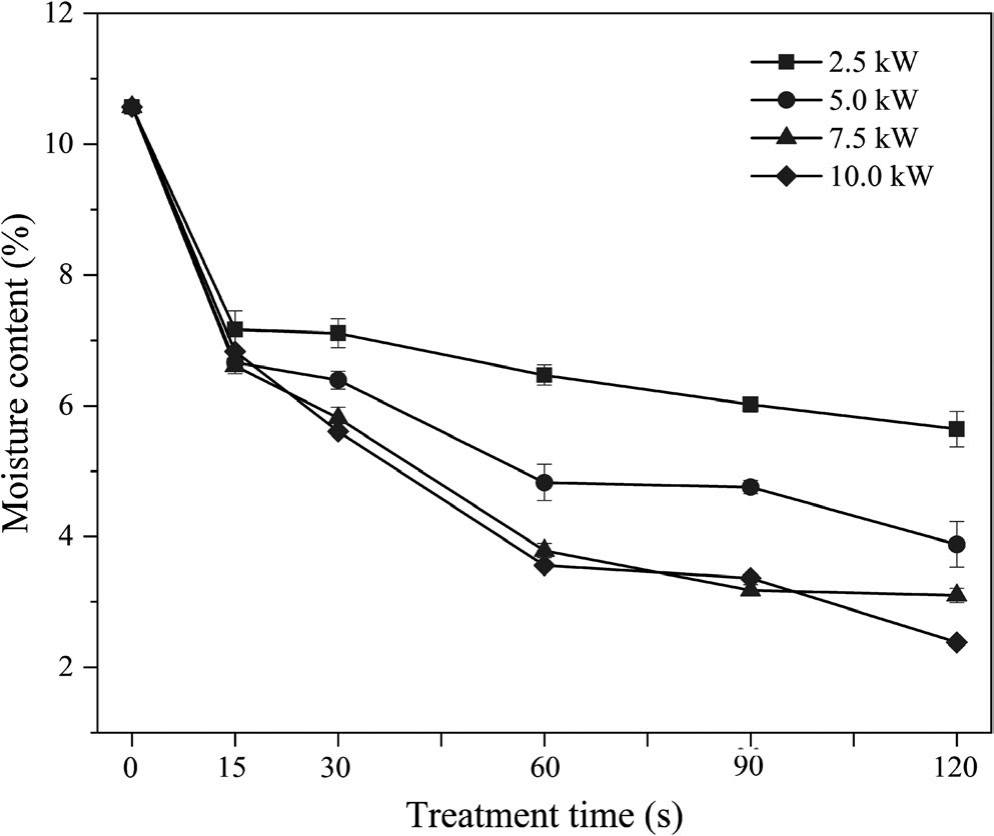
Effect of the microwave treatment on the moisture content of the FWB

As shown in Figure 1, the moisture content of FWB decreased with the increase of MW power and treatment time. MW power contributed much more for the reduction of water content when treatment time was more than 15 s. The moisture evaporation caused by MW treatment led to the reduction of water content of FWB (Chandrasekaran et al., 2013; Cheng et al., 2020). When treated at 7.5 kW for 90 s, the moisture content of the FWB initially decreased from 10.56% to 3.10% and subsequently kept a relatively constant level. The effect of MW treatment at 10.0 kW on the moisture content of the FWB was similar to that at 7.5 kW from 0 to 90 s, while a significant decline was observed when the FWB was treated at 10.0 kW for 120 s. This indicated that simultaneously increasing MW treatment power and time would further reduce the moisture content, which was similar to the changes reported by Zhang et al., (2020). Lower moisture content is beneficial for the safe storage of grain fractions, as water is necessary for microbial reproduction and enzymatic activities (Liu et al., 2020; Zhang et al., 2020). However, too low moisture content would lead to scorching during heat treatment, indicating the MW power and treatment time should not be excessive (Guiné et al., 2015).

### 3.2 Effect of MW treatment on the color values of the FWB

The influences of various MW treatment conditions on the color of FWB were presented in Table 1. The control had L*, a*, and b* values of 80.73, 4.80 and 18.90, respectively. All color values (L*, a*, b*) were significantly affected by the MW treatment. The FWB samples treated for 15 s at all treatment powers exhibited significantly higher L* values but lower a* and b* values compared with the control. As the treatment time increased, the L* values decreased while the a* and b* values increased in comparison with the control. This trend was in agreement with a report by Zhang et al., (2020). At low MW intensity, the main reaction inside the FWB was pigment degradation, which induced an increase in the L* value and decreases in the a* and b* values. With the increase of MW power and treatment time, nonenzymatic browning (Maillard reaction and caramelization reaction) occurred due to high temperature and the presence of proteins and reducing sugars in FWB, thereby resulting in a decrease in the L* value and increases in the a* and b* values.

**TABLE 1:** Effect of MW treatment on the color of the FWB

Power/kW	Time/s	Color			
		L*	a*	b*	*ΔE*
Control		80.73 ± 0.06 cd	4.80 ± 0.00^i^	18.90 ± 0.00^k^	—
2.5	15	81.33 ± 0.17^ab^	4.60 ± 0.08^j^	18.65 ± 0.17^kl^	0.68 ± 0.24^gh^
	30	78.57 ± 0.15^kl^	6.23 ± 0.06^a^	21.40 ± 0.10^cd^	3.60 ± 0.18^ab^
	60	78.80 ± 0.20^jk^	6.20 ± 0.10^a^	21.43 ± 0.15^b‐d^	3.48 ± 0.26^b^
	90	79.83 ± 0.41^gh^	5.30 ± 0.16^e^	20.00 ± 0.42^g^	1.51 ± 0.59^e^
	120	80.97 ± 0.25^bc^	4.97 ± 0.06^gh^	19.37 ± 0.21^ij^	0.60 ± 0.14^h^
5	15	81.50 ± 0.10^a^	4.50 ± 0.00^j^	18.53 ± 0.06^l^	0.91 ± 0.07^f–h^
	30	79.13 ± 0.25^ij^	5.90 ± 0.10^b^	21.13 ± 0.12^de^	2.96 ± 0.23^c^
	60	78.53 ± 0.32^kl^	6.27 ± 0.06^a^	21.73 ± 0.15^ab^	3.88 ± 0.29^ab^
	90	80.23 ± 0.06^ef^	5.13 ± 0.06^f^	19.87 ± 0.21^gh^	1.14 ± 0.21^e–g^
	120	80.83 ± 0.21^cd^	4.83 ± 0.06^hi^	19.27 ± 0.21^j^	0.43 ± 0.18^h^
7.5	15	81.53 ± 0.23^a^	4.57 ± 0.06^j^	18.67 ± 0.06^kl^	0.87 ± 0.24^f–h^
	30	79.17 ± 0.12^ij^	5.73 ± 0.06^c^	20.87 ± 0.06^ef^	2.68 ± 0.12^cd^
	60	78.30 ± 0.17^l^	6.33 ± 0.05^a^	21.75 ± 0.14^a^	4.05 ± 0.14^a^
	90	80.77 ± 0.12^cd^	4.87 ± 0.06^hi^	19.63 ± 0.06^hi^	0.75 ± 0.05^gh^
	120	80.53 ± 0.15^de^	4.80 ± 0.10^i^	19.77 ± 0.31^gh^	0.90 ± 0.32^f–h^
10	15	81.68 ± 0.28^a^	4.50 ± 0.00^j^	18.48 ± 0.05^l^	1.09 ± 0.23^e–g^
	30	79.48 ± 0.35^hi^	5.58 ± 0.15^d^	20.63 ± 0.26^f^	2.28 ± 0.40^d^
	60	78.63 ± 0.23^kl^	6.20 ± 0.00^a^	21.63 ± 0.06^a–c^	3.72 ± 0.08^ab^
	90	80.2 ± 0.17^e–g^	5.10 ± 0.00^fg^	19.80 ± 0.10^gh^	1.09 ± 0.12^e–g^
	120	79.93 ± 0.42^fg^	5.10 ± 0.17^fg^	19.87 ± 0.15^gh^	1.31 ± 0.39^ef^

Note

All data are expressed as the mean ± the standard deviation (*n* = 3). Mean in a column with different lowercase letters differ significantly (*p* < .05).

The maximum total color difference (*Δ*E) during the MW treatment was only 4.05. Moreover, significant decreases in browning were observed at all MW powers for 90 s and 120 s during the MW treatment, which were characterized by higher L* values and lower a*, b* and *Δ*E values than those at 30 s and 60 s. The MW treatment might be an effective approach to reduce undesirable impacts of nonenzymatic browning without drastically altering the color of the raw FWB (Zhang et al., 2020).

### 3.3 Effect of MW treatment on the lipase activity of the FWB

**FIGURE 2:**
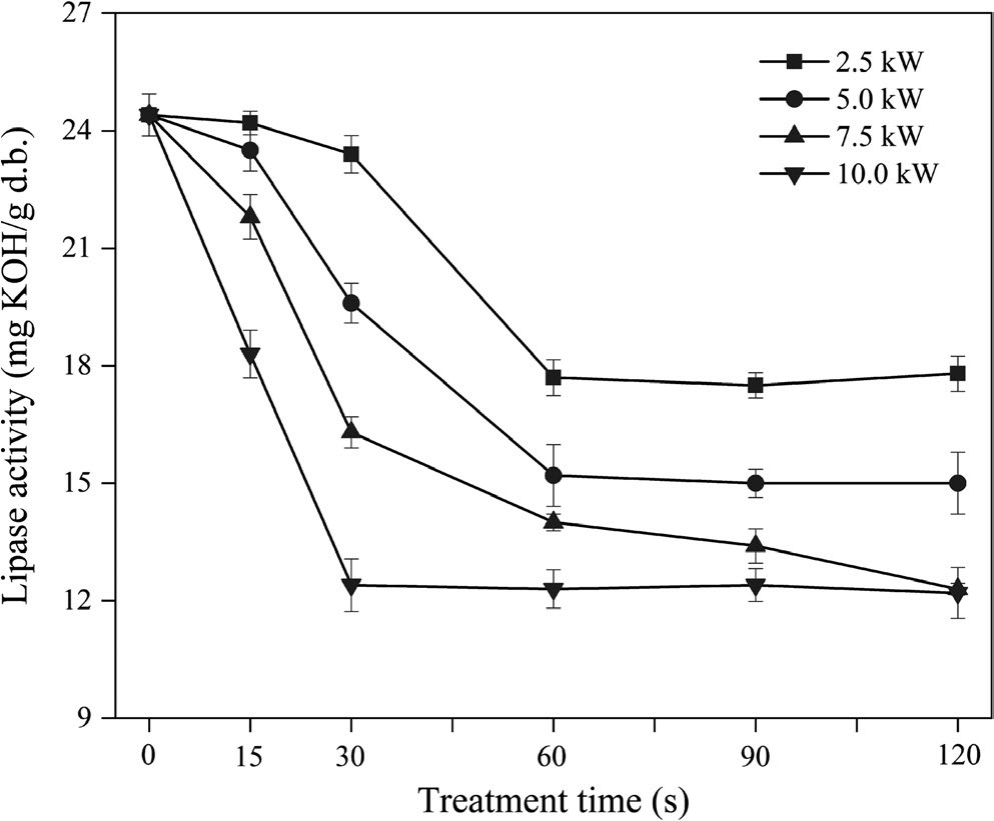
Effect of microwave treatment on the lipase activity of the FWB

Measuring the alteration of lipase activity was a convenient and practicable way to evaluate the stabilization efficacy of stabilization methods (Yu et al., 2020). The results of different MW treatments on the lipase activity were shown in Figure 2. The lipase activity of the raw FWB was 24.4 mg KOH/g d.b. Lipase was significantly inactivated after MW treatment at all powers and treatment times. The lipase activity of FWB treated with 2.5 kW for 60 s, 5.0 kW for 60 s, 7.5 kW for 120 s and 10.0 kW for 30 s decreased by 27%, 43%, 50% and 49%, respectively. Over time, the lipase activity of FWB initially decreased substantially and subsequently leveled off or decreased slightly, which was in agreement with a report of Rose et al., (2008). Moreover, the higher the MW power was, the lower the lipase activity. From the results presented, it is evident that MW power and treatment time were crucial and the former contributed more for inactivating lipase, which was similar to a previous report (Zhang et al., 2020).

Lipase was unstable and easy to be inactivated at high temperatures (Sharma et al., 2017). Thermal effects played a key role in inactivation of lipase by MW treatment (Chen et al., 2017) The temperature of materials treated by MW was influenced by moisture content since water is a satisfactory absorber of microwaves (Chandrasekaran et al., 2013). Thus, lipase activity significantly decreased at first and then leveled off due to the decrease of moisture content of FWB with the increase of treatment time. However, none of these treatments could completely inactivated lipase (the highest rate was 52%). The crude enzymes that are extracted from the treated FWB might contain phospholipase, esterase, or carbohydrase, which are present in the wheat germ, and some have higher thermal tolerance and are difficult to completely deactivate (Erika et al., 2010).

Similar lipase activity of FWB was obtained after treatment at 7.5 kW for 120 s and 10.0 kW for 30 s, which were close to the lowest lipase activity obtained in this study. In addition, the FWB sample treated at 7.5 kW for 120 s exhibited less color variation and lower moisture content than that at 10.0 kW for 30 s. Therefore, 7.5 kW for 120 s was selected as the optimal treatment conditions for stabilization of FWB.

### 3.4 Effect of MW treatment on the FAA content of the FWB

**FIGURE 3:**
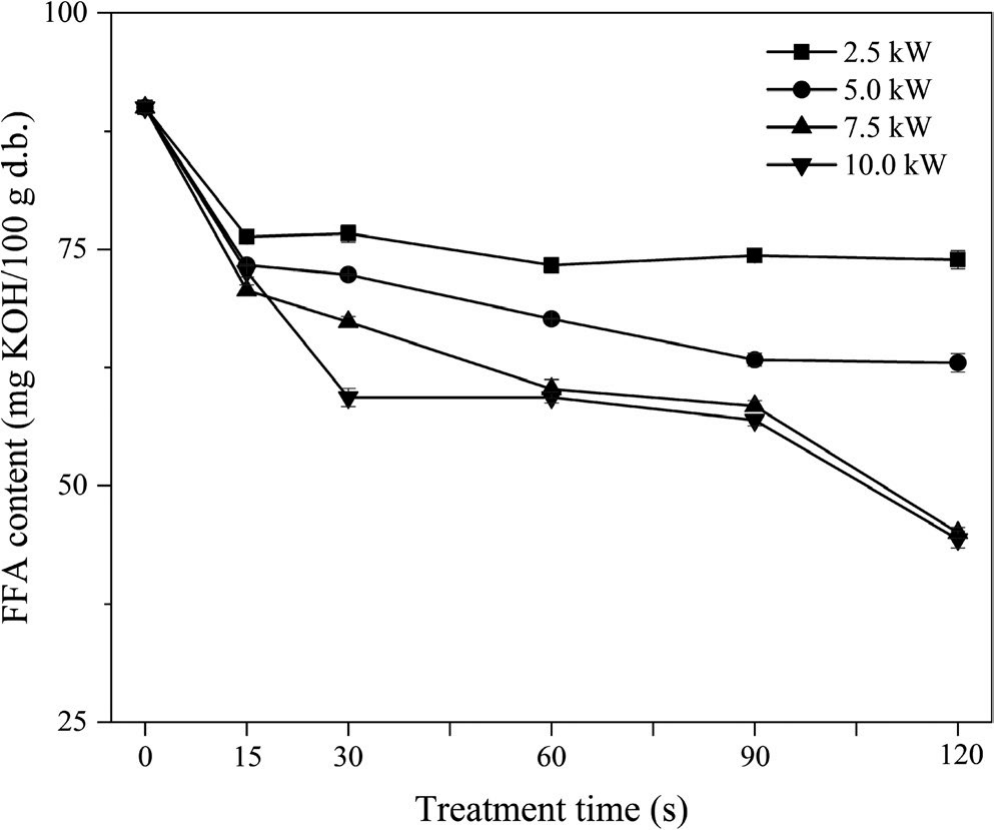
Effect of microwave treatment on the FFA content of the FWB

The FFA content is used as an indicator of the degree of hydrolytic rancidity of wheat bran. The FFA contents of FWB samples were determined immediately after grinding. The results are presented in Figure 3. The FFA content of the raw FWB was 90 mg KOH/100 g d.b., which was lower than the maximum value (120 mg KOH/100 g d.b.) that is considered acceptable for human consumption. A similar result was obtained by Hu et al., (2018), who reported that the FFA content of raw wheat bran was 81.87 mg KOH/100 g d.b.

Compared to the raw FWB, lower FFA contents were observed in all treated FWB samples. The FFA content decreased with the increase of treatment time and was significantly influenced by the MW power. The higher the MW power (2.5 ~ 7.5 kW), the lower the FFA contents after treatment for the same period of time. The effect of MW treatment at 10.0 kW on the FFA content of the FWB was similar to that at 7.5 kW. The lowest FFA content was 44.3 mg KOH/100 g d.b. (approximately 49% that of the raw FWB) after MW treatment for 120 s. Zhao et al., (2007) reported that the FFA content of rice decreased with an increase of MW energy consumption. Similarly, Adebowale et al., (2020) reported that the FFA content of MW‐treated whole grain sorghum flour was approximately 50% lower than that of an untreated sample. More FFAs might be degraded into hydroperoxides at higher MW power (Malheiro et al., 2011). These unstable peroxides decomposed swiftly into volatile compounds, which reduced the FFA content of the samples (Doblado‐Maldonado, 2012).

### 3.5 Effects of MW treatment on the TPC and antioxidant activity of the FWB

**FIGURE 4:**
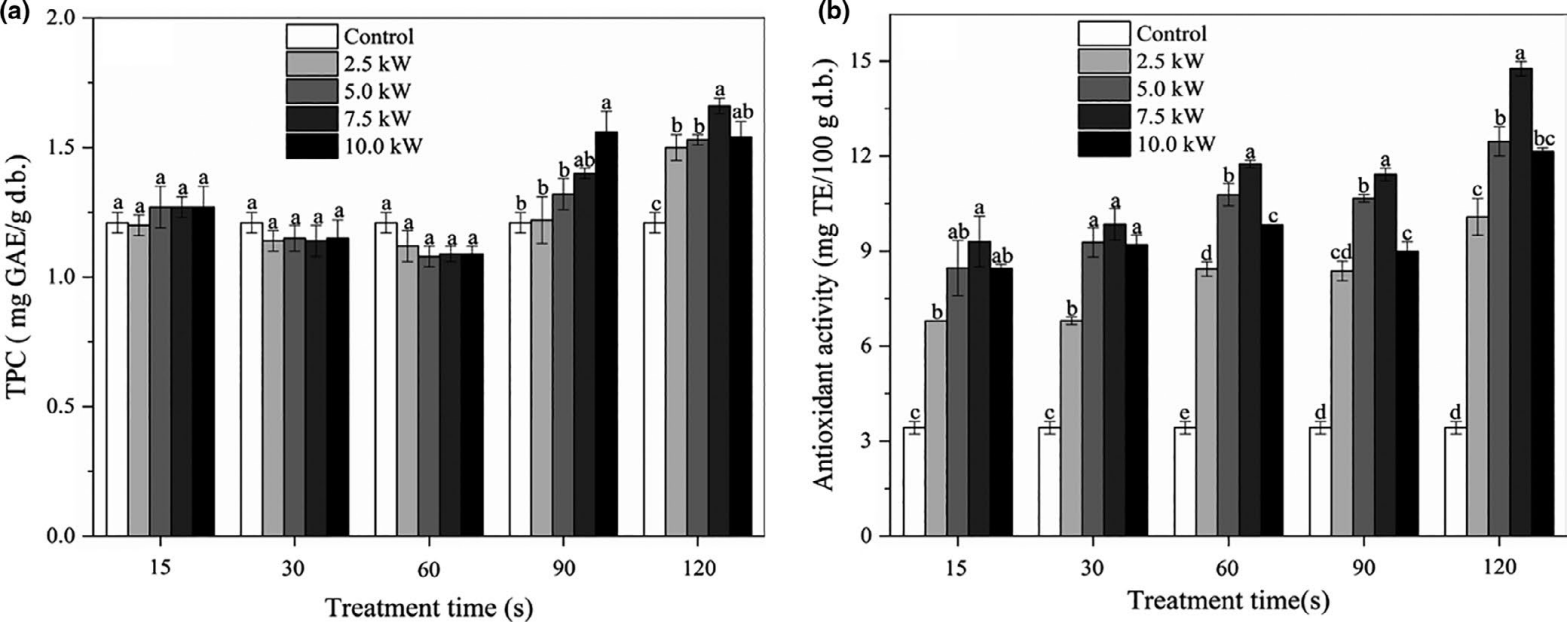
Effects of MW treatment on the TPC (a) and antioxidant activity (b) of the FWB

The phenolic compounds in wheat bran have demonstrated potent antioxidant and free radical scavenging properties (Abozed et al., 2014). The TPC of FWB samples are presented in Figure 4a. The TPC of the control was 1.21 mg GAE/g d.b. Compared to the control, the TPC of the FWB samples were slightly influenced by the MW treatment during the period of 0 ~ 60 s at all powers (*p* >.05). Significant increases in the TPC were observed after MW treatment for 90 s and 120 s (*p* <.05), regardless of the MW power. The increase in TPC of the treated FWB ranged between 9% and 37%. The maximum value (1.66 mg GAE/g d.b.) was obtained from the FWB sample treated at 7.5 kW for 120 s. The results demonstrated that the TPC of FWB increased as a result of MW stabilization. Similar observations were reported by Saji et al., (2020) and Irakli et al., (2018) wherein stabilized rice bran had higher free and bound TPC compared to nonstabilized rice bran. During stabilization, the MW heating might lead to breakdown of cell wall structure and the liberation of the bound phenolic compounds. On the contrary, Rose et al., (2008) reported that MW treatment with 1.0 kW for 120 s had no significant effect on the TPC of wheat bran. This might indicate that the MW power contributed more than the treatment time for the release of phenolic compounds. However, too high treatment temperature and too long treatment time would lead to the loss of phenolics via thermal degradation (Rodchuajeen et al., 2016).

The effect of MW treatment on the antioxidant activity of the FWB was presented in Figure 4b. The antioxidant activity of the control was 3.43 mg TE/100 g d.b. The antioxidant activity of the MW‐treated FWB significantly increased with increasing treatment time compared to that of the control (*p* <.05). The highest value of the antioxidant activity was 14.76 mg TE/100 g d.b. for the FWB sample that was treated at 7.5 kW for 120 s, which was approximately 4.3 times higher than that of the control. This could be explained by the strong correlation between the TPC and the antioxidant capacity (Verma et al., 2009). Nevertheless, the trend of antioxidant activity of the FWB was not completely consistent with that of TPC. This demonstrated that other nonphenolic antioxidants (such as Maillard reaction products) might also contribute to the antioxidant activity of the FWB (Liu et al., 2016). Therefore, MW treatment may be an effective technology that could simultaneously stabilize the FWB and increase its antioxidant activities.

### 3.6 Moisture and FFA contents of the stabilized FWB during storage

**FIGURE 5:**
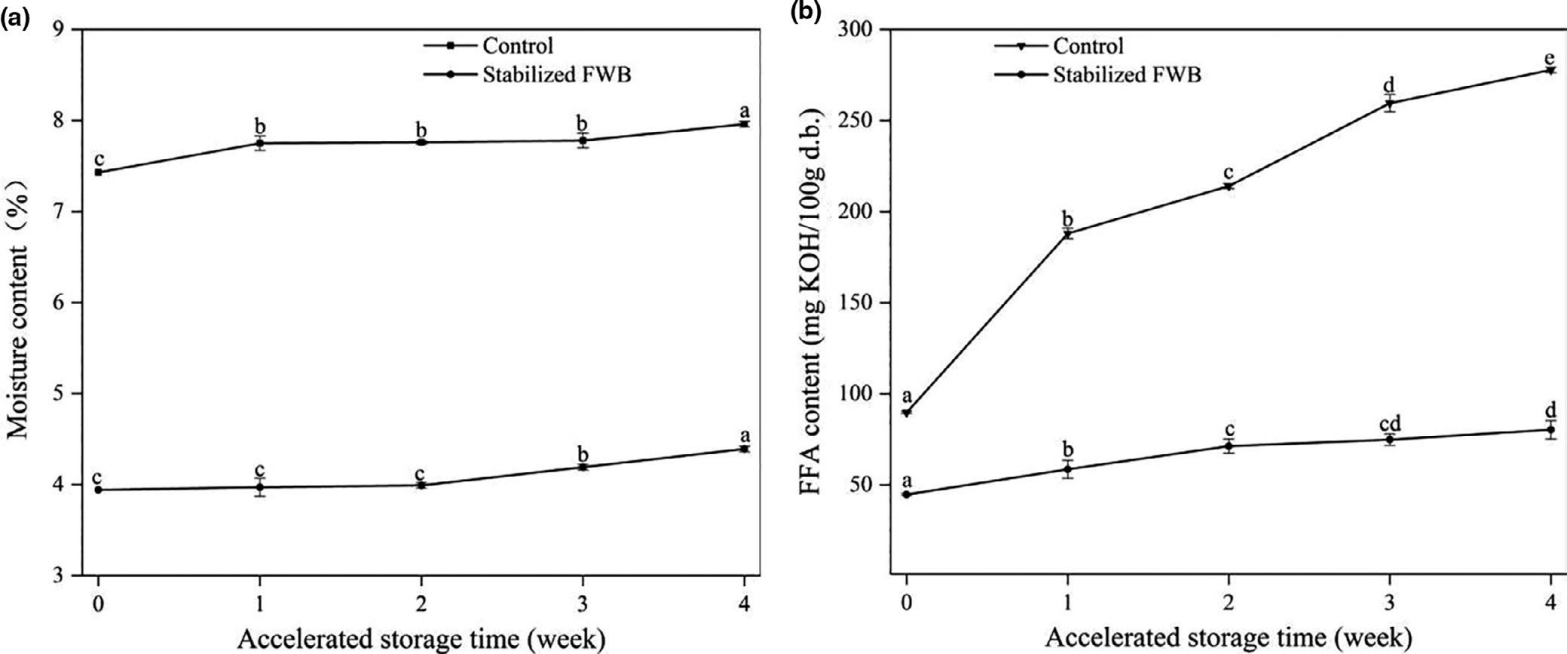
Changes in the moisture and FFA contents of the raw FWB and stabilized FWB during 4 weeks of storage in an oven at 45°C (the raw FWB was used as the control)

Based on the above results, 7.5 kW for 120 s was identified as the optimal MW treatment conditions to prepare the sample for the accelerated storage test. The moisture and FFA contents are shown in Figure 5 for the MW‐treated FWB samples and the control during storage. With the increase of the storage time, the moisture contents of the control and stabilized FWB slightly increased from 7.43% and 3.94% to 7.96% and 4.39%, respectively. All of them satisfy the moisture content standard of edible wheat bran (≤12%).

FFA content continuously increased as the storage time increased (Figure 5). Lipase exhibits maximum activity in wheat at about 17% moisture content (Rose & Pike, 2006). However, lipase activity of MW‐treated samples (3% ~ 4% moisture) was about 50% of the control FWB sample (Figure 2). Hydrolysis of lipids by lipase resulted in the release of FFAs. The increase of FFA content for the treated FWB sample was much lower than those for control because of the lower moisture content and lipase activity. As shown in Figure 5, the FFA content in the treated FWB after storage for 4 weeks at 45°C was 80.3 mg KOH/100 g d.b., which was more suitable for human consumption than the control (278 mg KOH/100 g d.b.). The MW treatment could effectively retard FFA formation during storage of FWB. These results are in agreement with the findings reported by Rose et al., (2008) and Patil et al., (2016).

## 4 CONCLUSIONS

The effects of microwave treatment on the stability and antioxidant capacity of functional wheat bran were investigated. MW treatment significantly decreased the FFA content, moisture content, and lipase activity of the FWB and improved the TPC and antioxidant activity of the FWB without drastically altering the color. The MW power contributed more than the treatment time, and 7.5 kW for 120 s was identified as the optimal MW treatment condition. The results of this study show that MW treatment can increase the storage stability and antioxidant capacity of the FWB and could provide a scientific foundation for application of MW in industrial production of FWB.

## CONFLICT OF INTEREST

The authors declare no conflict of interest.

## ETHICAL APPROVAL

Ethics approval was not required for this research.

## References

[fsn32230-bib-0001] AACC International (2010). Approved Methods of the American Association of Cereal Chemists International (10th ed.). The Association: St. Paul.

[fsn32230-bib-0002] Abozed, S. S. , El‐kalyoubi, M. , Abdelrashid, A. , & Salama, M. F. (2014). Total phenolic contents and antioxidant activities of various solvent extracts from whole wheat and bran. Annals of Agricultural Science, 59, 63–67. 10.1016/j.aoas.2014.06.009

[fsn32230-bib-0003] Adebowale, O. J. , Taylor, J. R. N. , & de Kock, H. L. (2020). Stabilization of wholegrain sorghum flour and consequent potential improvement of food product sensory quality by microwave treatment of the kernels. LWT ‐ Food Science and Technology, 132, 109827. 10.1016/j.lwt.2020.109827

[fsn32230-bib-0004] Bagdi, A. , Szabó, F. , Gere, A. , Kókai, Z. , Sipos, L. , & Tömösközi, S. (2014). Effect of aleurone‐rich flour on composition, cooking, textural, and sensory properties of pasta. LWT ‐ Food Science and Technology, 59, 996–1002. 10.1016/j.lwt.2014.07.001

[fsn32230-bib-0005] Bagdi, A. , Tóth, B. , Lőrincz, R. , Szendi, S. , Gere, A. , Kókai, Z. , Sipos, L. , & Tömösközi, S. (2016). Effect of aleurone‐rich flour on composition, baking, textural, and sensory properties of bread. LWT ‐ Food Science and Technology, 65, 762–769. 10.1016/j.lwt.2015.08.073

[fsn32230-bib-0006] Barnes, P. , & Galliard, T. (1991). Rancidity in cereal products. Lipid Technology, 3, 23–28.

[fsn32230-bib-0007] Blandino, M. , Sovrani, V. , Marinaccio, F. , Reyneri, A. , Rolle, L. , Giacosa, S. , Locatelli, M. , Bordiga, M. , Travaglia, F. , Coisson, J. D. , & Arlorio, M. (2013). Nutritional and technological quality of bread enriched with an intermediated pearled wheat fraction. Food Chemistry, 141, 2549–2557. 10.1016/j.foodchem.2013.04.122 23870994

[fsn32230-bib-0008] Boukid, F. , Folloni, S. , Ranieri, R. , & Vittadini, E. (2018). A compendium of wheat germ: Separation, stabilization and food applications. Trends in Food Science & Technology, 78, 120–133. 10.1016/j.tifs.2018.06.001

[fsn32230-bib-0009] Brouns, F. , Hemery, Y. , Price, R. , & Anson, N. M. (2012). Wheat aleurone: Separation, composition, health aspects, and potential food use. Critical Reviews in Food Science and Nutrition, 52, 553–568. 10.1080/10408398.2011.589540 22452734

[fsn32230-bib-0010] Chandrasekaran, S. , Ramanathan, S. , & Basak, T. (2013). Microwave food processing—A review. Food Research International, 52, 243–261. 10.1016/j.foodres.2013.02.033

[fsn32230-bib-0011] Cheli, F. , Campagnoli, A. , Ventura, V. , Brera, C. , Berdini, C. , Palmaccio, E. , & Dell'Orto, V. (2010). Effects of industrial processing on the distributions of deoxynivalenol, cadmium and lead in durum wheat milling fractions. LWT ‐ Food Science and Technology, 43, 1050–1057. 10.1016/j.lwt.2010.01.024

[fsn32230-bib-0012] Chen, Z. W. , Li, Y. L. , Wang, L. K. , Liu, S. Y. , Wang, K. K. , Sun, J. , & Xu, B. (2017). Evaluation of the possible non‐thermal effect of microwave radiation on the inactivation of wheat germ lipase. Journal of Food Process Engineering, 40, e12506. 10.1111/jfpe.12506

[fsn32230-bib-0013] Cheng, Y. , Wang, W. , Zhang, R. , Zhai, X. , & Hou, H. (2020). Effect of gelatin bloom values on the physicochemical properties of starch/gelatin–beeswax composite films fabricated by extrusion blowing. Food Hydrocolloids, 113, 106466. 10.1016/j.foodhyd.2020.106466

[fsn32230-bib-0014] Doblado‐Maldonado, A. F. (2012). New Technologies for Whole Wheat Processing: Addressing Milling and Storage Issues. University of Nebraska‐Lincoln.

[fsn32230-bib-0015] Erika, L. , Attila, K. , György, V. , & Miklós, N. (2010). Influence of microwave radiation on lipase and xanthine oxidase activity in milk. Magyar Allatorvosok Lapja, 132, 728–734. 10.1109/WCSN.2010.5712295

[fsn32230-bib-0016] Guiné, R. P. F. , Almeida, C. F. F. , Correia, P. M. R. , & Mendes, M. (2015). Modelling the influence of origin, packing and storage on water activity, colour and texture of almonds, hazelnuts and walnuts using artificial neural networks. Food and Bioprocess Technology, 8, 1113–1125. 10.1007/s11947-015-1474-3

[fsn32230-bib-0017] Hu, Y. M. , Wang, L. J. , & Li, Z. G. (2018). Superheated steam treatment on wheat bran: Enzymes inactivation and nutritional attributes retention. LWT ‐ Food Science and Technology, 91, 446–452. 10.1016/j.lwt.2018.01.086

[fsn32230-bib-0018] Irakli, M. , Kleisiaris, F. , Mygdalia, A. , & Katsantonis, D. (2018). Stabilization of rice bran and its effect on bioactive compounds content, antioxidant activity and storage stability during infrared radiation heating. Journal of Cereal Science, 80, 135–142. 10.1016/j.jcs.2018.02.005

[fsn32230-bib-0019] Liu, H. , Liu, X. , Zhao, F. , Liu, Y. , Liu, L. , Wang, L. , Geng, C. , & Huang, P. (2020). Preparation of a hydrophilic and antibacterial dual function ultrafiltration membrane with quaternized graphene oxide as a modifier. Journal of Colloid and Interface Science, 562, 182–192. 10.1016/j.jcis.2019.12.017 31838354

[fsn32230-bib-0020] Liu, L. , Li, J. , Yue, F. , Yan, X. , Wang, F. , Bloszies, S. , & Wang, Y. F. (2018). Effects of arbuscular mycorrhizal inoculation and biochar amendment on maize growth, cadmium uptake and soil cadmium speciation in cd‐contaminated soil. Chemosphere, 194, 495–503. 10.1016/j.chemosphere.2017.12.025 29241123

[fsn32230-bib-0021] Liu, L. Y. , Zhao, M. L. , Liu, X. Y. , Zhong, K. , Tong, L. T. , Zhou, X. R. , & Zhou, S. M. (2016). Effect of steam explosion‐assisted extraction on phenolic acid profiles and antioxidant properties of wheat bran. Journal of the Science of Food and Agriculture, 96, 3484–3491. 10.1002/jsfa.7532 26572692

[fsn32230-bib-0022] Malheiro, R. , Casal, S. , Ramalhosa, E. , & Alberto, J. (2011). Microwave Heating: A Time Saving Technology or a Way to Induce Vegetable Oils Oxidation? In S. Grundas (Ed.), Advances in induction and microwave heating of mineral and organic materials (pp. 597–614). InTech.

[fsn32230-bib-0023] Patil, S. S. , Kar, A. , & Mohapatra, D. (2016). Stabilization of rice bran using microwave: Process optimization and storage studies. Food and Bioproducts Processing, 99, 204–211. 10.1016/j.fbp.2016.05.002

[fsn32230-bib-0024] Reynolds, A. , Mann, J. , Cummings, J. , Winter, N. , Mete, E. , & Te Morenga, L. (2019). Carbohydrate quality and human health: A series of systematic reviews and meta‐analyses. The Lancet, 393, 434–445. 10.1016/S0140-6736(18)31809-9 30638909

[fsn32230-bib-0025] Rodchuajeen, K. , Niamnuy, C. , Charunuch, C. , Soponronnarit, S. , & Devahastin, S. (2016). Stabilization of rice bran via different moving bed drying methods. Drying Technology, 34, 1854–1867. 10.1080/07373937.2016.1236345

[fsn32230-bib-0026] Rose, D. J. , Ogden, L. V. , Dunn, M. L. , & Pike, O. A. (2008). Enhanced lipid stability in whole wheat flour by lipase inactivation and antioxidant retention. Cereal Chemistry, 85, 218–223. 10.1094/CCHEM-85-2-0218

[fsn32230-bib-0027] Rose, D. , & Pike, O. (2006). A simple method to measure lipase activity in wheat and wheat bran as an estimation of storage quality. Journal of the American Oil Chemists' Society, 83, 415e419. 10.1007/s11746-006-1220-0

[fsn32230-bib-0028] Saji, N. , Schwarz, L. J. , Santhakumar, A. B. , & Blanchard, C. L. (2020). Stabilization treatment of rice bran alters phenolic content and antioxidant activity. Cereal Chemistry, 97(2), 281–292. 10.1002/cche.10243

[fsn32230-bib-0029] Sharma, P. , Sharma, N. , Pathania, S. , & Handa, S. (2017). Purification and characterization of lipase by Bacillus methylotrophicus PS3 under submerged fermentation and its application in detergent industry. Journal of Genetic Engineering & Biotechnology, 15, 369–377. 10.1016/j.jgeb.2017.06.007 30647675 PMC6296573

[fsn32230-bib-0030] Verma, B. , Hucl, P. , & Chibbar, R. N. (2009). Phenolic acid composition and antioxidant capacity of acid and alkali hydrolysed wheat bran fractions. Food Chemistry, 116, 947–954. 10.1016/j.foodchem.2009.03.060

[fsn32230-bib-0031] Wang, N. F. , Hou, G. G. , Kweon, M. , & Lee, B. (2016). Effects of particle size on the properties of whole‐grain soft wheat flour and its cracker baking performance. Journal of Cereal Science, 69, 187–193. 10.1016/j.jcs.2016.03.010

[fsn32230-bib-0032] Wu, J. Y. , McClements, D. J. , Chen, J. , Liu, W. , Luo, S. J. , & Liu, C. M. (2016). Improvement in storage stability of lightly milled rice using superheated steam processing. Journal of Cereal Science, 71, 130–137. 10.1016/j.jcs.2016.08.006

[fsn32230-bib-0033] Xu, B. , Zhou, S. L. , Miao, W. J. , Gao, C. , Cai, M. J. , & Dong, Y. (2013). Study on the stabilization effect of continuous microwave on wheat germ. Journal of Food Engineering, 117, 1–7. 10.1016/j.jfoodeng.2013.01.031

[fsn32230-bib-0034] Xu, M. , Hou, G. G. , Ma, F. Y. , Ding, J. Z. , Deng, L. Z. , Kahraman, O. , Niu, M. , Trivettea, K. , Lee, B. , Wu, L. , & Baik, B. K. (2020). Evaluation of aleurone flour on dough, textural, and nutritional properties of instant fried noodles. LWT ‐ Food Science and Technology, 126, 109294. 10.1016/j.lwt.2020.109294

[fsn32230-bib-0035] Xu, Y. , Yang, J. , Du, L. , Li, K. , & Zhou, Y. (2018). Association of whole grain, refined grain, and cereal consumption with gastric cancer risk: A meta‐analysis of observational studies. Food Science & Nutrition, 7(113–120), 256–265, 10.1002/fsn3.878 30680179 PMC6341150

[fsn32230-bib-0036] Yadav, D. N. , Anand, T. , Kaur, J. , & Singh, A. K. (2012). Improved storage stability of pearl millet flour through microwave treatment. Agricultural Research, 1, 399–404. 10.1007/s40003-012-0040-8

[fsn32230-bib-0037] Yu, C. W. , Hu, Q. R. , Wang, H. W. , & Deng, Z. Y. (2020). Comparison of 11 rice bran stabilization methods by analyzing lipase activities. Journal of Food Processing and Preservation, 44(4), e14370. 10.1111/jfpp.14370

[fsn32230-bib-0038] Zhang, Y. K. , Tang, N. , Shi, L. , Miao, Y. X. , Liu, X. , Ge, X. H. , Cheng, Y. Q. , & Zhang, X. Q. (2020). Characterization and comparison of predominant aroma compounds in microwave‐treated wheat germ and evaluation of microwave radiation on stability. Journal of Cereal Science, 93, 102942. 10.1016/j.jcs.2020.102942

[fsn32230-bib-0039] Zhao, S. M. , Xiong, S. B. , Qiu, C. G. , & Xu, Y. L. (2007). Effect of microwaves on rice quality. Journal of Stored Products Research, 43, 496–502. 10.1016/j.jspr.2007.02.002

[fsn32230-bib-0040] Zhu, S. Y. , Zheng, Z. J. , Peng, H. W. , Sun, J. , Zhao, X. E. , & Liu, H. W. (2020). Quadruplex stable isotope derivatization strategy for the determination of panaxadiol and panaxatriol in foodstuffs and medicinal materials using ultra high performance liquid chromatography tandem mass spectrometry. Journal of Chromatography A, 1616, 460794. 10.1016/j.chroma.2019.460794 31870574

